# Up-regulation of fibrinogen-like protein 2 in porcine endothelial cells by xenogeneic CD40 signal

**DOI:** 10.1080/19768354.2018.1433718

**Published:** 2018-02-01

**Authors:** Bumrae Cho, Inho Choi, Eun Mi Lee, Sunghoon Hurh, Byeong Chun Lee, Curie Ahn

**Affiliations:** a Biotechnology Research Institute, Mgenplus Co., Ltd., Seoul, Republic of Korea; b Department of Theriogenology and Biotechnology, College of Veterinary Medicine, Seoul National University, Seoul, Republic of Korea; c Research Institute for Veterinary Science, Seoul National University, Seoul, Republic of Korea; d Department of Pharmaceutical Engineering, College of Life and Health Sciences, Hoseo University, Asan, Republic of Korea; e Center for Medical Innovation, Biomedical Research Institute, Seoul National University Hospital, Seoul, Korea; f Graduate School of Translational Medicine, Seoul National University College of Medicine, Seoul, Republic of Korea; g Department of Internal Medicine, Seoul National University College of Medicine, Seoul, Republic of Korea

**Keywords:** Fibrinogen-like protein 2, CD40-CD40L interaction, endothelial cells, xenotransplantation, thrombosis

## Abstract

Acute humoral xenograft rejection (AHXR), characterized by thrombin generation and endothelial cell activation, should be overcome for the success of xenotransplantation. Fibrinogen-like protein 2 (fgl2) expressed on endothelial cells can convert prothrombin to thrombin directly, which indicates that the induced fgl2 expression in activated endothelial cells can contribute to thrombosis. In xenotransplant condition, the interaction between human CD40L and porcine endothelial CD40 can activate endothelial cells. In this study, we investigated the effect of endothelial cell activation through the interaction between human CD40L and porcine CD40 on fgl2 expression and its function as a direct prothrombinase. We found that CD40 stimulation up-regulated fgl2 expression as well as its enzymatic activity in porcine endothelial cells. Moreover, functional studies using knock-down system showed that the major factor converting human prothrombin to thrombin is fgl2 protein expressed on porcine endothelial cells. Overall, this study demonstrates that fgl2 expression can be induced by xenogeneic CD40 signal on endothelial cells and contribute to thrombin generation.

## 1. Introduction

Organ shortage in transplantation can be solved by xenotransplantation, the transplantation of organs from different species. Hyperacute xenograft rejection (HXR), an obstacle observed early after transplantation, is considered to be overcome by the generation of α1,3-galactosyltransferase knockout (GalT KO) pigs (Phelps et al. 2003; Kolber-Simonds et al. 2004). Indeed, in pig-to-nonhuman primate xenotransplantation using GalT KO pig, the heterotropic heart and kidney xenograft survived for 6 and 3 months, respectively, indicating that HXR could be successfully overcome (Shimizu et al. 2008; Ekser et al. 2009). However, acute humoral xenograft rejection (AHXR) still remains to be conquered for successful xenotransplantation. One of the causes of GalT KO pig xenograft failure could be thrombotic microangiopathy, which is caused by injury or activation of endothelial cells. Several studies have shown that the exposure of endothelial cells to xenoreactive natural antibodies, complements, platelets, immune cells, or cytokines, causes a loss in the natural anticoagulant state, and the acquisition of procoagulant state through a combination of morphological changes and altered gene expressions (Bach et al. 1994; Robson et al. 1995). In addition, porcine endothelial cells are capable of generating thrombin directly from the human prothrombin, in the absence of other coagulation factors (Jurd et al. 1996; Siegel et al. 1997). While there are increasing evidences pointing to the activation of graft endothelial cells as a major cause of thrombosis, the putative molecular mechanism that may promote coagulation in xenografts remains to be solved (Cowan 2007; Cooper et al. 2015, 2016).

Fibrinogen-like protein 2 (fgl2) has direct prothrombinase activity without a classical prothrombinase complex (Chan et al. 2002; Yang and Hooper^2013^). Previous studies have shown that fgl2 is involved in experimental xenograft rejection. This is achieved by mediating coagulation, fibrin deposition, and microthrombus formation; typical pathological changes resulting in acute humoral xenograft rejection (Ghanekar et al. 2004; Ning et al. 2005; Cooper et al. 2015). Another study suggested that allograft rejection correlates with fgl2 expression and can be prevented by the administration of neutralizing anti-CD154 (Cluster of Differentiation 40L; CD40L) monoclonal antibody (Hancock et al. 2004), although the mechanism by which fgl2 inhibition occurs was not elucidated.

We previously reported that porcine endothelial cells can be activated by xenogeneic interaction between human CD40L (hCD40L) and porcine CD40 (pCD40) (Choi et al. 2008). In this study, based on our previous report, we have investigated potential prothrombotic microenvironments mediated by fgl2 molecule on endothelial cells activated by the xenogeneic interaction between hCD40L and pCD40. We found that fgl2 expression was increased by CD40 stimulation in porcine endothelial cells by human CD40L expressing cells (Jurkat D1.1 or THP-1), or agonistic anti-CD40 antibody in RNA and protein levels. Functional studies using knock-down system clearly showed that fgl2 expressed on endothelial cells plays a key role for converting human prothrombin to thrombin. Overall, this study demonstrates that xenogeneic CD40 signal can contribute to thrombin generation by up-regulating fgl2 expression on endothelial cells.

## 2. Materials and methods

### 2.1. Cells and reagents

MPN3, a miniature pig aortic endothelial cell line (Kim et al. 2005), Jurkat D1.1, a human T cell line, and THP-1, a human monocytic cell line, were used. MPN3 was cultured or co-cultured with Jurkat D1.1 or THP-1 cells in Dulbecco's modified essential media (DMEM) (Wellgene, Korea) supplemented with 10% fetal bovine serum (FBS) (Gibco, Life Technologies, CA), and 1% antibiotic/antimycotic (Gibco) at 37°C containing 5% CO_2_. Neutralizing anti-CD154 antibody (Clone 24-31) was purchased from Ancell (MN, USA). Recombinant human TNF-α was purchased from eBioscience (CA, USA) and the agonistic anti-CD40 antibody (Clone 82111) was purchased from R&D system (MN, USA).

### 2.2. Reverse transcription-polymerase chain reaction (RT-PCR)

The expression of *fgl2* transcripts were analyzed by semi-quantitative RT-PCR with *fgl2*-specific primers (forward: 5′-AGCTGATGACAGCAGAGTTAGAG-3′, reverse: 5′-AGTGATCATACAAGGCATAGAGC-3′) and *GAPDH (glyceraldehyde 3-phosphate dehydrogenase*)-specific primers (forward: 5′- ACAGCCTCAAGATCATCAGCAAT-3′, reverse: 5′- AGGAAATGAGCTTGACAAAGTGG-3′) to allow equal amounts of cDNA templates. First, total RNA was isolated from MPN3 cells using TRIzol reagent (Life Technology, CA) and reverse-transcribed using MMLV reverse transcriptase (Enzynomics, Korea) and 15 mer oligo-dT (Enzynomics, Korea). Using cDNA as a template, PCR was performed according to the following protocols: (1) 94°C for 5 min; (2) 26 cycles at 94°C for 30 s, 58°C for 30 s, and 72°C for 1 min; and (3) 72°C for 10 min before cooling to 4°C. Ten microliters of PCR products were loaded on a 1.5% agarose gel containing ethidium bromide and visualized by ultraviolet irradiation.

### 2.3. Western blot analysis

Cell lysates were prepared using RIPA buffer (50 mM Tris-HCl [pH 7.4], 150 mM NaCl, 1% Nonidet P-40, 0.1% sodium dodecyl sulfate [SDS], and 0.5% sodium deoxycholate) containing protease inhibitors (Sigma-Aldrich, CA). Total proteins were quantified using the Bradford Protein Assay kit (Pierce Biotechnology, IL) according to the manufacturer's instructions. 20 μg of proteins were then separated by 10% SDS polyacrylamide gel electrophoresis and transferred to PVDF (polyvinylidene difluoride membranes; GE Healthcare, UK) membrane. Western blots were performed with suitable antibodies and chemiluminescent signal detection reagent (AbClone, Korea). Anti-fgl2 monoclonal antibody (Clone 6D9) was purchased from Abnova (Taiwan) and anti-α-tubulin monoclonal antibody was purchased from AbFrontier (Korea). HRP-conjugated goat anti-mouse IgG antibody was purchased from Santa Cruz biotechnology (TX, USA).

### 2.4. Immunofluorescence microscopy

Immunofluorescence microscopy was performed as described previously (Lee et al. 2005). Briefly, the attached MPN3 cells were fixed and incubated at room temperature (RT). The cells were then incubated with anti-fgl2 antibody and then with FITC-conjugated mouse IgG antibody (Santa Cruz biotechnology, TX, USA). These cells were mounted on glass slides using Fluoroshield™ with 4′,6-diamidino-2-phenylindole (DAPI) (Sigma Aldrich). Images were obtained using a LSM5 PASCAL fluorescence microscope (Zeiss, Germany).

### 2.5. Thrombin generation assay

Thrombin generation assay was performed as reported in a previous study (Ghanekar et al. 2004). Briefly, cells were harvested, washed three times with cold reaction buffer [20 mM HEPES (4-(2-hydroxyethyl)-1-piperazineethanesulfonic acid) (pH 7.4), 150 mM sodium chloride, and 5 mM calcium chloride] and resuspended in reaction buffer at a population of 5 × 10^5^ cells/mL. A total of 1 × 10^5^ cells were mixed with an equal volume of human prothrombin (American Diagnostica, Germany) in reaction buffer to give a final prothrombin concentration of 1 μM. For additional negative controls, the same number of cells were incubated with reaction buffer alone, and prothrombin was incubated with reaction buffer alone. The reaction was carried out for 20 min at 37°C in triplicate for each experiment. To measure the level of the generated thrombin, 125 µL of cold assay buffer [50 mM Tris (pH 8.3), 227 mM sodium chloride, 1% bovine serum albumin (BSA), and 1% sodium azide] was added to each reaction. Following centrifugation at 14,000 rpm for 5 min to pellet cells, 145 µL of supernatant from each reaction mixture was transferred to a flat-bottom 96-well plate. 15 µL of Chromozym TH^TM^ (Roche, Switzerland), a chromogenic substrate of human thrombin, was added to each well, and the plate incubated at room temperature. Optical Density (OD) was measured at 405 nm and thrombin activity of each sample was calculated by comparison with the absorbance values of a thrombin series (Sigma-Aldrich).

### 2.6. Knock-down experiments using shRNA expression vectors

Short hairpin RNA (shRNA) sequences for porcine *fgl2* and *CD40* mRNA were selected, confirmed to be specific for the target genes, synthesized chemically (Table 1), and inserted into the shRNA-expressing vector (pSNU6-1) (Choi et al. 2005). Knock-down efficiency was confirmed by introduction of shRNA vector into COS-7 (shRNA against CD40; shCD40) or MPN3 (shRNA against fgl2; shFgl2) cells. Then, MPN3 cells were transfected with each shRNA expression vector and treated with agonistic anti-CD40 antibody (5 μg/mL) for 48 h. After incubation, thrombin generation assay was performed.

**Table 1.** Oligomers used for the construction of shRNA expression vectors.

Name	Sequences
shFgl2-1 F	5’-GCT GAA GCT GTC GAA CTG GTA GAT CTG ACC AGT TCG ACA GCT TCA GCT TTT TT-3’
shFgl2-1 R	5’-AGC TAA AAA AGC TGA AGC TGT CGA ACT GGT CAG ATC TAC CAG TTC GAC AGC TTC AGC GGC C-3’
shFgl2-2 F	5’-TTA CGT TGA TAA CAA GGT GTA GAT CTG ACA CCT TGT TAT CAA CGT AAT TTT TT-3’
shFgl2-2 R	5’-AGC TAA AAA ATT ACG TTG ATA ACA AGG TGT CAG ATC TAC ACC TTG TTA TCA ACG TAA GGC C-3’
shCD40-1 F	5’-AAC AGA CAC CAC TTG TGT GTA GAT CTG ACA CAC AAG TGG TGT CTG TTT TTT TT-3’
shCD40-1 R	5’-AGC TAA AAA AAA CAG ACA CCA CTT GTG TGT CAG ATC TAC ACA CAA GTG GTG TCT GTT GGC C-3’
shCD40-2 F	5’-GGC CCT GCA CCC TAA GAC TTA GAT CTG AAG TCT TAG GGT GCA GGG CCT TTT TT-3’
shCD40-2 R	5’-AGC TAA AAA AGG CCC TGC ACC CTA AGA CTT CAG ATC TAA GTC TTA GGG TGC AGG GCC GCC C-3’

### 2.7. Statistical analysis

Statistically significant differences were identified by Student's *t*-test using SPSS 18.0 (SPSS Inc., NY); *p* < 0.05 values were considered to be statistically significant.

## 3. Results

### 3.1. Fgl2 expression induced by CD40 signal

**Figure 1.**
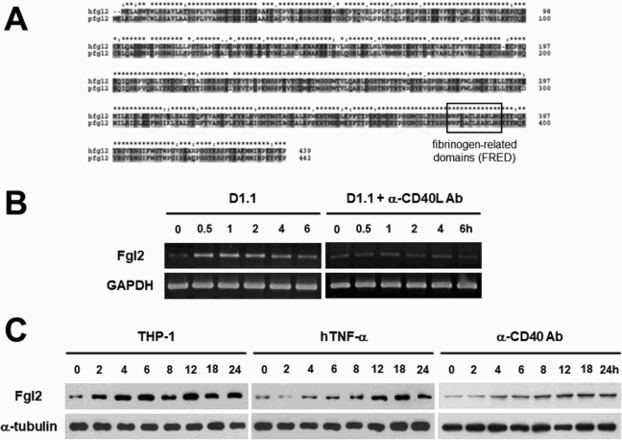
Up-regulation of fibrinogen-like protein 2 (fgl2) in porcine endothelial cells. (A) Sequence analysis shows homology (90%) between of human and porcine fgl2 proteins and fibrinogen-related domain (FRED) is conserved in the C-terminus of fgl2. (B) The expression of *fgl2* mRNA in porcine endothelial cells stimulated by Jurkat T cell line (D1.1) pre-treated with or without neutralizing anti-CD40L antibody was analyzed by semi-quantitative RT-PCR. *GAPDH* gene was used as a quantitative control. (C) The expression of fgl2 protein was measured by western blot analysis. Fgl2 expression was increased time-dependently by co-culture with human monocytic cell line (THP-1), pro-inflammatory cytokine (TNF-α), or agonistic anti-CD40 antibody. α-tubulin was detected as a quantitative control.

The amino acid sequences of human and porcine fgl2 protein were compared using the Clustal X program {Jeanmougin et al. 1998} and a high sequence homology (90%) was observed. In addition, the amino acid sequences of fibrinogen-related domain (FRED) in the C-terminus of fgl2 were conserved, which means the molecular compatibility of fgl2 molecule across species (Figure 1A). To determine whether the fgl2 expression in porcine endothelial cells could be modulated by interactions with human cells, MPN3, a porcine endothelial cell line, was co-cultured with Jurkat D1.1, a human T cell line. Semi-quantitative RT-PCR results showed that the induction of *fgl2* mRNA in MPN3 cells occurred as quickly as 30 min. Interestingly, when MPN3 cells were co-cultured with Jurkat D1.1 cells pre-incubated with neutralizing anti-CD40L antibody, *fgl2* expression was not affected (Figure 1B). Following confirmation of porcine fgl2 up-regulation by CD40L-expressing human T cells, MPN3 cells were stimulated with another cell line expressing CD40L (THP-1, a human monocytic cell line), or with human TNF-α (20 ng/mL), a strong pro-inflammatory cytokine activating porcine endothelial cells [17]. To investigate whether the CD40 signal was solely responsible for the up-regulation of fgl2, the MPN3 cells were treated with an agonistic anti-CD40 antibody (clone 82111). Western blot analysis showed that the expression of fgl2 was up-regulated at 4 h after treatment with TNF-α or an agonistic anti-CD40 antibody. On the other hand, fgl2 expression was induced very rapidly when MPN3 cells were co-cultured with THP-1 cells (Figure 1C). These results indicate that xenogenic CD40 signal can induce the expression of fgl2 in porcine endothelial cells.

### 3.2. Up-regulation of fgl2 expression on endothelial cells by CD40 signal

**Figure 2.**
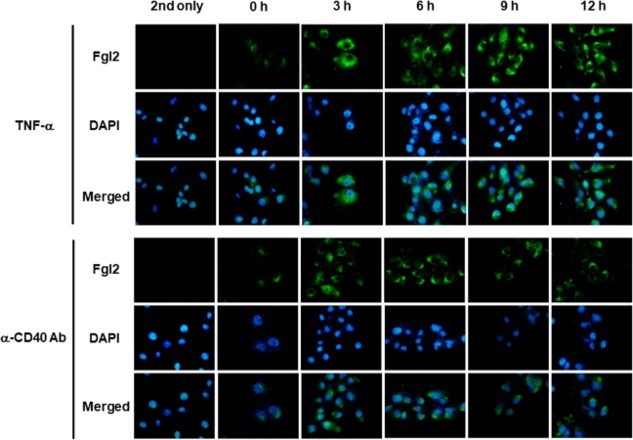
Up-regulation of fgl2 on porcine endothelial cells. Immunofluorescence microscopy was performed to identify fgl2 protein expression on the endothelial cell surface. Fgl2 protein expression on the endothelial cell surface was up-regulated at an early time by TNF-α or agonistic anti-CD40 antibody and decreased after 9–12 h. DAPI staining was carried out to identify the cell nuclei.

Next, immunofluorescence microscopy analysis was performed to investigate the expression of fgl2 on endothelial cells and showed that fgl2 expression on MPN3 cell surface was increased at early time after treatment with agonistic anti-CD40 antibody as well as TNF-α (Figure 2). Fgl2 expression on endothelial cells was induced from 3 h after treatment of TNF-α and anti-CD40 antibody, which means that fgl2 expression can be up-regulated on endothelial cells stimulated with CD40 signal as well as with a pro-inflammatory cytokine.

### 3.3. Up-regulation of fgl2 prothrombinase activity by CD40 signal

**Figure 3.**
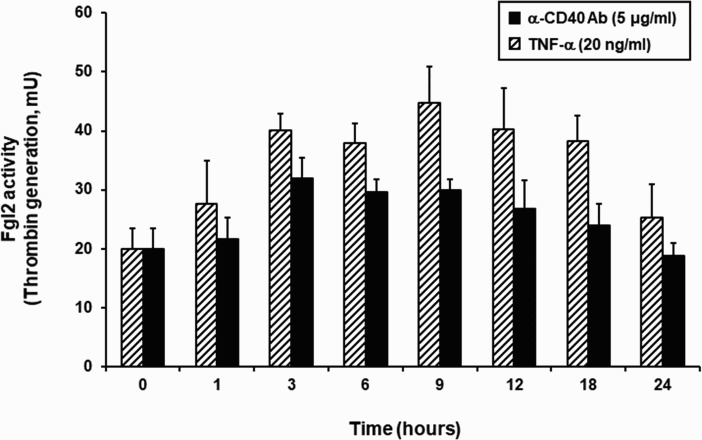
Generation of human thrombin by up-regulated fgl2 in porcine endothelial cells. Thrombin generation assay was performed to validate the enzyme activity of fgl2. After treatment of TNF-α (20 ng/mL) or agonistic anti-CD40 antibody (5 μg/mL), the absorbance was measured using a chromogenic substrate (Chromozym TH), and the amount of thrombin was calculated by comparison with a standard curve. Striped bars (▨) indicate TNF-α group (*N* = 6) and black bars (▪) indicate agonistic anti-CD40 antibody group (*N* = 6).

The prothrombinase activity of fgl2 was investigated using the thrombin generation assay. MPN3 cells were stimulated with an agonistic anti-CD40 antibody, or TNF-α as a control, harvested at various time points (0, 1, 3, 6, 9, 12, 18 and 24 h), and analyzed for the prothrombinase enzyme activity which regulates the generation of thrombin from human prothrombin (Figure 3). The results showed a 1.5 fold increase in the prothrombinase activity of fgl2 from 3 to 9 h after stimulation using an agonistic anti-CD40 antibody. Pro-inflammatory TNF-α, used as a positive control, increased fgl2 function in MPN3 cells. Negative control reactions, missing either prothrombin or MPN3 cells, did not generate any prothrombinase activity, confirming that the assay was not contaminated with thrombin (data not shown). These results indicate that prothrombinase activity of fgl2 in endothelial cells can be induced by stimulating CD40 signal as well as TNF-α.

### 3.4. Knock-down effect on prothrombinase activity of fgl2

**Figure 4.**
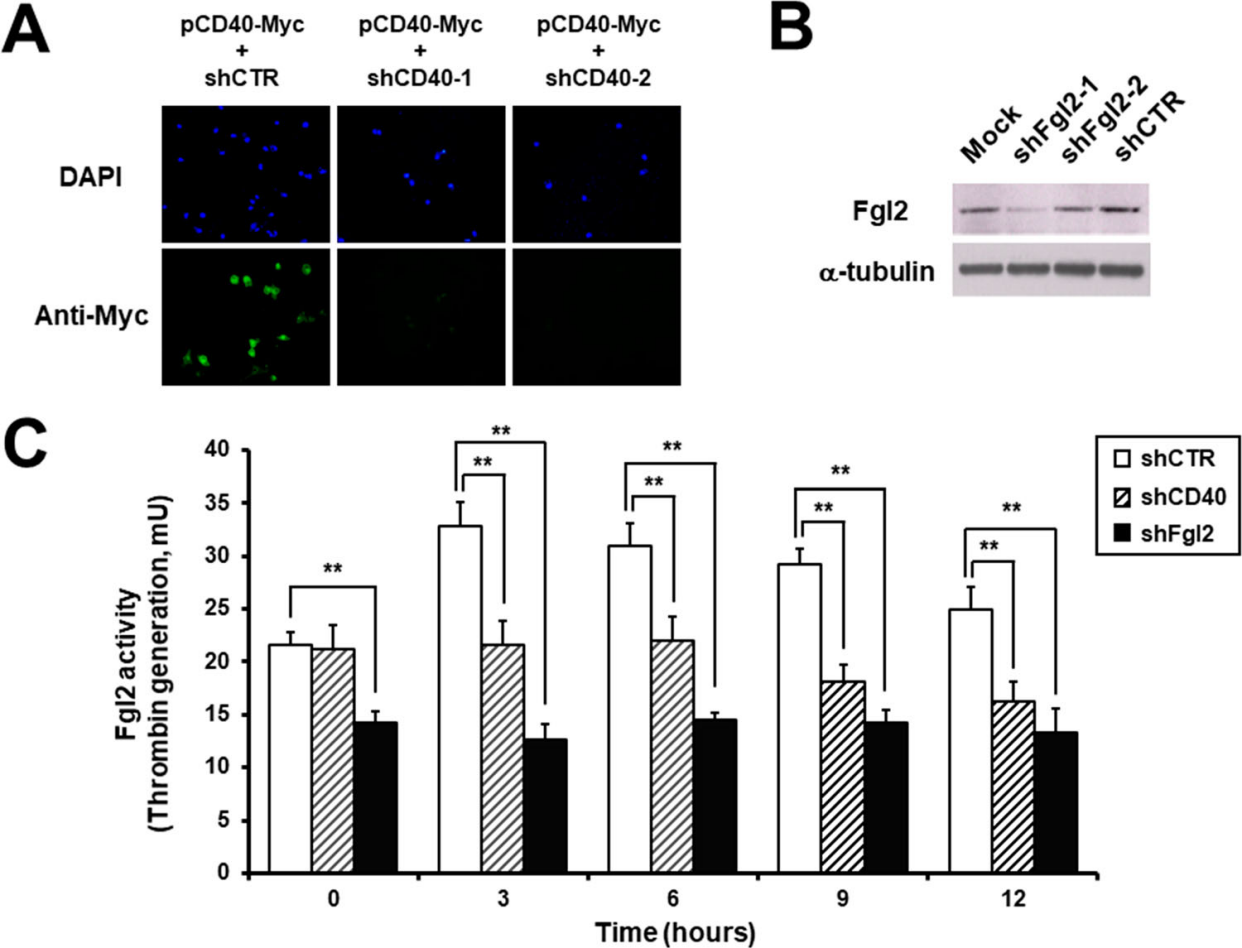
*CD40* or *fgl2* knock-down effect on fgl2 activity. (A) To investigate the efficacy of CD40 knock-down system, porcine CD40 expression vector and shCD40 expression vector were co-transfected into COS-7 cells, and CD40 was detected by immunofluorescence microscopy. DAPI staining was performed to identify cell nuclei. (B) shFgl2 expression vector was transfected into endothelial cells and fgl2 expression was analyzed by western blot. α-tubulin was detected as a quantitative control. (C) Thrombin generation assay was performed to measure the effect of knock-down system on the prothrombinase activity of fgl2. Although agonistic anti-CD40 antibody upregulated the enzymatic activity (white bars), the thrombin generation was not increased by CD40 signal stimulation in knock-down cells (shCD40, striped bars; shFgl2, black bars) (*N* = 5, ** *p* < 0.01).

Knock-down system expressing shRNA was used to validate the role of CD40 molecule in the up-regulation of fgl2. The suppressive effects of shCD40 and shFgl2 were verified by immunofluorescence microscopy and western blot analysis, respectively (Figure 4A, 4B). At 48 h after transfection, porcine endothelial cells were stimulated with agonistic anti-CD40 antibody and thrombin generation assay was performed (Figure 4C). MPN3 cells expressing control shRNA (shCTR) were used as a negative control and showed a similar basal activity to that of non-transfected MPN3 cells (Figure 3) for thrombin generation. Similar to the previous result (Figure 3), fgl2 prothrombinase activity was up-regulated by CD40 signal during 3–9 h and reduced from 12 h. Interestingly, when compared to the control MPN3 cells (shCTR), fgl2 prothrombinase activity was not induced by the CD40 signal in CD40 knock-down MPN3 cells (*p* < 0.01). Moreover, prothrombinase activity in MPN3 cells expressing shFgl2 was much lower than that in shCD40-expressing MPN3 cells, even before stimulation (Figure 4C). These functional studies using knock-down system indicate that thrombin generation can be increased by fgl2 molecule whose expression is up-regulated by stimulating CD40 signal in endothelial cells.

## 4. Discussion

Thrombosis and fibrin deposition are known as key processes in acute humoral xenograft rejection (AHXR). When porcine endothelial cells are activated, the expressions of co-stimulatory molecules, various cytokines/chemokines, or pro-coagulant molecules such as tissue factor are up-regulated. Indeed, in pig-to-baboon xenotransplantation using transgenic pig for human decay-accelerating factor (hDAF), the transplanted porcine kidney showed features of AHXR such as thrombotic microangiopathy, interstitial hemorrhage, intravascular thrombosis, and fibrin deposition (Ghanekar et al. 2002; Zhong et al. 2003; Miwa et al. 2015). This susceptibility of porcine endothelial cells to coagulation and thrombosis may be induced due to the molecular incompatibility of anti-coagulant molecules between two species (Cowan & d’Apice 2008; Miwa et al. 2015). Meanwhile, there is a report indicating the importance of fgl2 molecule for thrombin generation in acute humoral xenograft rejection (Cooper et al. 2016).

In this study, we first checked that fgl2 proteins share 90% homology between human and porcine, and have a conserved fibrinogen-related domain (FRED) in the C terminus of the protein, which indicates the possible involvement of porcine fgl2 in the formation of a prothrombotic microenvironment in xenotransplantation (Figure 1A). Indeed, a previous study showed that porcine fgl2 can convert human prothrombin to functional thrombin (Ghanekar et al. 2004). After confirming the molecular homology of fgl2 proteins, we employed various methods to stimulate CD40 signal in endothelial cells. In the co-culture experiment using Jurkat D1.1 cells expressing high levels of cell surface CD40L (Choi et al. 2008), the neutralizing anti-CD40 antibody completely blocked the induction of *fgl2* mRNA expression in endothelial cells. This result indicates that the increase of *fgl2* transcripts in MPN3 cells stimulated with Jurkat D1.1 cells is mainly dependent on CD40 signal (Figure 1B). Moreover, when MPN3 cells were treated with agonistic anti-CD40 antibody alone, fgl2 expression was increased (Figure 1C). Interestingly, fgl2 expression was induced rapidly in MPN3 cells when cells were stimulated by THP-1 cells, which means that human monocytes may express variable other factors stimulating porcine endothelial cells beyond co-stimulatory signals.

Immunofluorescence microscopic analysis showed that fgl2 expression on MPN3 cell surface was increased at early time after treatment of TNF-α or agonistic anti-CD40 antibody, and reduced after 9–12 h (Figure 2). However, western blot analysis showed that fgl2 protein was still expressed up to 24 h after treatment with TNF-α or agonistic anti-CD40 antibody (Figure 1C). This discordance in data may be explained by the discrepancy in subcellular localization of fgl2 protein, which means that the functional fgl2 showing prothrombinase activity is detected only on the cell surface membrane. In addition, we performed prothrombinase assay to investigate the variation of fgl2 activity by CD40 signal and found that the enzyme activity of fgl2 was increased at 3–9 h after stimulation with agonistic anti-CD40 antibody and decreased after 12 h (Figure 3). Therefore, these results demonstrate that fgl2 may be biologically active only on the cell surface membrane, although its expression is still detected in endothelial cells.

Next, knock-down systems were used to verify the effect of CD40 signal on fgl2 up-regulation (Figure 4). For this purpose, we employed an shRNA expression system (Choi et al. 2005). After confirming the inhibitory effect of each shRNA, the prothrombinase activity of fgl2 was tested. Compared to shCTR (negative control), shCD40 completely inhibited the generation of thrombin. We also noticed that prothrombinase activity in MPN3 cells expressing shFgl2 was very low, before as well as after CD40 stimulation. These results clearly show that surface fgl2 molecule plays a key role in thrombin generation, even in unstimulated endothelial cells, and that CD40 signal can provoke the up-regulation of fgl2. In addition, these results may propose a mechanism for the down-regulation of fgl2 expression by neutralizing anti-CD154 (CD40L) monoclonal antibody in allotransplantation model (Ghanekar et al. 2004).

We previously reported that xenogeneic interaction between human CD40L and porcine CD40 can activate porcine endothelial cells, and increase the expression of adhesion molecules or chemokines via the NF-κB signaling pathway (Choi et al. 2008). Combined with the results of this previous study, here, we demonstrate that the activation of porcine endothelial cells by CD40 signal can up-regulate fgl2 expression and its prothrombinase activity, and suggest that genetic modification targeting *CD40* or *fgl2* should be considered to avoid thrombotic microenvironment causing xenotransplant rejection.

## References

[CIT0001] Bach FH, Robson SC, Ferran C, Winkler H, Millan MT, Stuhlmeier KM, Vanhove B, Blabcely ML, Van Der Werf WJ, Hofer E, et al. 1994 Endothelial cell activation and thromboregulation during xenograft rejection. Immunol Rev. 141:5–30. doi: 10.1111/j.1600-065X.1994.tb00870.x 7868157

[CIT0002] Chan CW, Chan MW, Liu M, Fung L, Cole EH, Leibowitz JL, Marsden PA, Clark DA, Levy GA. 2002 Kinetic analysis of a unique direct prothrombinase, fgl2, and identification of a serine residue critical for the prothrombinase activity. J Immunol. 168:5170–5177. doi: 10.4049/jimmunol.168.10.5170 11994472

[CIT0003] Choi I, Cho BR, Kim D, Miyagawa S, Kubo T, Kim JY, Park CG, Hwang WS, Lee JS, Ahn C. 2005 Choice of the adequate detection time for the accurate evaluation of the efficiency of siRNA-induced gene silencing. J Biotechnol. 120:251–261. doi: 10.1016/j.jbiotec.2005.06.014 16095743

[CIT0004] Choi I, Kim SD, Cho B, Kim D, Park D, Koh HS, Kim BY, Kim JY, Yang J, Ahn C. 2008 Xenogeneic interaction between human CD40L and porcine CD40 activates porcine endothelial cells through NF-kappaB signaling. Mol Immunol. 45:575–580. doi: 10.1016/j.molimm.2007.06.161 17675236

[CIT0005] Cooper DK, Ekser B, Ramsoondar J, Phelps C, Ayares D. 2016 The role of genetically engineered pigs in xenotransplantation research. J Pathol. 238:288–299. doi: 10.1002/path.4635 26365762 PMC4689670

[CIT0006] Cooper DK, Ekser B, Tector AJ. 2015 Immunobiological barriers to xenotransplantation. Int J Surg. 23:211–216. doi: 10.1016/j.ijsu.2015.06.068 26159291 PMC4684773

[CIT0007] Cowan PJ. 2007 Coagulation and the xenograft endothelium. Xenotransplantation. 14:7–12. doi: 10.1111/j.1399-3089.2006.00368.x 17214700

[CIT0008] Cowan PJ, d’Apice AJ. 2008 The coagulation barrier in xenotransplantation: incompatibilities and strategies to overcome them. Curr Opin Organ Transplant. 13:178–183. doi: 10.1097/MOT.0b013e3282f63c74 18685300

[CIT0009] Ekser B, Rigotti P, Gridelli B, Cooper DK. 2009 Xenotransplantation of solid organs in the pig-to-primate model. Transpl Immunol. 21:87–92. doi: 10.1016/j.trim.2008.10.005 18955143

[CIT0010] Ghanekar A, Lajoie G, Luo Y, Yang H, Choi J, Garcia B, Cole EH, Greig PD, Cattral MS, Phillips MJ, et al. 2002 Improvement in rejection of human decay accelerating factor transgenic pig-to-primate renal xenografts with administration of rabbit antithymocyte serum. Transplantation. 74:28–35. doi: 10.1097/00007890-200207150-00006 12134095

[CIT0011] Ghanekar A, Mendicino M, Liu H, He W, Liu M, Zhong R, Phillips MJ, Levy GA, Grant DR. 2004 Endothelial induction of fgl2 contributes to thrombosis during acute vascular xenograft rejection. J Immunol. 172:5693–5701. doi: 10.4049/jimmunol.172.9.5693 15100314

[CIT0012] Hancock WW, Szaba FM, Berggren KN, Parent MA, Mullarky IK, Pearl J, Cooper AM, Ely KH, Woodland DL, Kim IJ, et al. 2004 Intact type 1 immunity and immune-associated coagulative responses in mice lacking IFN gamma-inducible fibrinogen-like protein 2. Proc Natl Acad Sci USA. 101:3005–3010. doi: 10.1073/pnas.0308369101 14976252 PMC365735

[CIT0013] Jeanmougin F, Thompson JD, Gouy M, Higgins DG, Gibson TJ. 1998 Multiple sequence alignment with Clustal X. Trends Biochem Sci. 23:403–405. doi: 10.1016/S0968-0004(98)01285-7 9810230

[CIT0014] Jurd KM, Gibbs RV, Hunt BJ. 1996 Activation of human prothrombin by porcine aortic endothelial cells--a potential barrier to pig to human xenotransplantation. Blood Coagul Fibrolysis. 7:336–343. doi: 10.1097/00001721-199604000-00008 8735141

[CIT0015] Kim D, Kim JY, Koh HS, Lee JP, Kim YT, Kang HJ, Hwang WS, Kim YB, Lee JS, Ahn C. 2005 Establishment and characterization of endothelial cell lines from the aorta of miniature pig for the study of xenotransplantation. Cell Biol Int. 29:638–646. doi: 10.1016/j.cellbi.2005.03.016 15950500

[CIT0016] Kolber-Simonds D, Lai L, Watt SR, Denaro M, Arn S, Augenstein ML, Betthauser J, Carter DB, Greenstein JL, Hao Y, et al. 2004 Production of alpha-1,3-galactosyltransferase null pigs by means of nuclear transfer with fibroblasts bearing loss of heterozygosity mutations. Proc Natl Acad Sci USA. 101:7335–7340. doi: 10.1073/pnas.0307819101 15123792 PMC409919

[CIT0017] Lee EM, Kim JY, Cho BR, Chung WK, Yoon BW, Kim SU, Lee BC, Hwang WS, Moon SY, Lee JS, et al. 2005 Down-regulation of MHC class I expression in human neuronal stem cells using viral stealth mechanism. Biochem Biophys Res Commun. 326:825–835. doi: 10.1016/j.bbrc.2004.11.106 15607744

[CIT0018] Miwa Y, Yazaki S, Iwamoto M, Suzuki S, Iwasaki K, Haneda M, Yamamoto K, Maruyama S, Onishi A, Kobayashi T. 2015 Functional difference between membrane-bound and soluble human thrombomodulin. Transplantation. 99:702–709. doi: 10.1097/TP.0000000000000571 25643141

[CIT0019] Ning Q, Sun Y, Han M, Zhang L, Zhu C, Zhang W, Guo H, Li J, Yan W, Gong F, et al. 2005 Role of fibrinogen-like protein 2 prothrombinase/fibroleukin in experimental and human allograft rejection. J Immunol. 174:7403–7411. doi: 10.4049/jimmunol.174.11.7403 15905589

[CIT0020] Phelps CJ, Koike C, Vaught TD, Boone J, Wells KD, Chen SH, Ball S, Specht SM, Polejaeva IA, Monahan JA, et al. 2003 Production of alpha 1,3-galactosyltransferase-deficient pigs. Science. 299:411–414. doi: 10.1126/science.1078942 12493821 PMC3154759

[CIT0021] Robson SC, Candinas D, Hancock WW, Wrighton C, Winkler H, Bach FH. 1995 Role of endothelial cells in transplantation (part 1 of 2). Int Arch Allergy Immunol. 106:305–314. doi: 10.1159/000236861 7719148

[CIT0022] Shimizu A, Hisashi Y, Kuwaki K, Tseng YL, Dor FJ, Houser SL, Robson SC, Schuurman HJ, Cooper DK, Sachs DH, et al. 2008 Thrombotic microangiopathy associated with humoral rejection of cardiac xenografts from alpha1,3-galactosyltransferase gene-knockout pigs in baboons. Am J Pathol. 172:1471–1481. doi: 10.2353/ajpath.2008.070672 18467706 PMC2408408

[CIT0023] Siegel JB, Grey ST, Lesnikoski BA, Kopp CW, Soares M, Schulte am Esch 2nd J, Bach FH, Robson SC. 1997 Xenogeneic endothelial cells activate human prothrombin. Transplantation. 64:888–896. doi: 10.1097/00007890-199709270-00017 9326416

[CIT0024] Yang G, Hooper WC. 2013 Physiological functions and clinical implications of fibrinogen-like 2: a review. World J Clin Infect Dis. 3:37–46. doi: 10.5495/wjcid.v3.i3.37 26161303 PMC4495006

[CIT0025] Zhong R, Luo Y, Yang H, Garcia B, Ghanekar A, Luke P, Chakrabarti S, Lajoie G, Phillips MJ, Katopodis AG, et al. 2003 Improvement in human decay accelerating factor transgenic porcine kidney xenograft rejection with intravenous administration of gas914, a polymeric form of alphaGAL. Transplantation. 75:10–19. doi: 10.1097/00007890-200301150-00003 12544864

